# Transcription factors that shape the mammalian pancreas

**DOI:** 10.1007/s00125-020-05161-0

**Published:** 2020-09-07

**Authors:** Rachel E. Jennings, Raphael Scharfmann, Willem Staels

**Affiliations:** 1 Division of Diabetes, Endocrinology & Gastroenterology, Faculty of Biology, Medicine & Health, University of Manchester, AV Hill Building, Oxford Road, Manchester, M13 9PT UK; 2 Endocrinology Department, Manchester University NHS Foundation Trust, Manchester, UK; 3 Institut Cochin, INSERM, U1016, CNRS, UMR8104, Université de Paris, 75014 Paris, France; 4 Beta Cell Neogenesis (BENE), Vrije Universiteit Brussel, Laarbeeklaan 103, 1090 Brussels, Belgium; 5 Department of Pediatrics, Division of Pediatric Endocrinology, University Hospital of Brussels, Jette, Belgium

**Keywords:** Development, Human, Islets of Langerhans, Mouse, Neonatal diabetes, NEUROG3, Neurogenin 3, Pancreas and duodenal homeobox 1, PDX1, Regulatory factor X6, Review, RFX6, Transcription factors

## Abstract

Improving our understanding of mammalian pancreas development is crucial for the development of more effective cellular therapies for diabetes. Most of what we know about mammalian pancreas development stems from mouse genetics. We have learnt that a unique set of transcription factors controls endocrine and exocrine cell differentiation. Transgenic mouse models have been instrumental in studying the function of these transcription factors. Mouse and human pancreas development are very similar in many respects, but the devil is in the detail. To unravel human pancreas development in greater detail, in vitro cellular models (including directed differentiation of stem cells, human beta cell lines and human pancreatic organoids) are used; however, in vivo validation of these results is still needed. The current best ‘model’ for studying human pancreas development are individuals with monogenic forms of diabetes. In this review, we discuss mammalian pancreas development, highlight some discrepancies between mouse and human, and discuss selected transcription factors that, when mutated, cause permanent neonatal diabetes.

**Electronic supplementary material:**

The online version of this article (10.1007/s00125-020-05161-0) contains a slide of the figure for download, which is available to authorised users.

**Figure:**
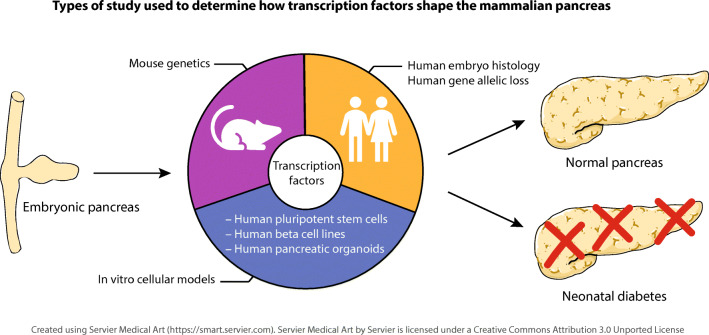
Graphical abstract

## Scope

During human pancreas development, the orchestration of differentiation fates and the acquisition of endocrine and exocrine cell identity depend on the controlled expression of unique sets of transcription factors. Transgenic mouse models in which transcription factor expression can be manipulated have been key tools in studies to improve our understanding of pancreas organogenesis. Most of what we have learnt in mice holds true for humans, based on transcription factor expression patterns at different developmental stages. In this review we will discuss some of the differences that have been described. New tools have become available for refined experiments in which the expression of transcription factors can be modulated in human models. Stem cells, for example, have rapidly become a new paradigm for modelling organ development and disease. However, the gold standard for determining the impact of transcription factor perturbation on human pancreas development remains human gene allelic loss in individuals presenting with monogenic forms of diabetes. In this review we do not exhaustively list all transcription factors important for pancreas development, but we specifically address selected transcription factors during human pancreas development, the consequences of transcription factor perturbations on murine and human pancreas development, and neonatal diabetes as a means of understanding pancreas development. We also provide some reflections on therapy in developmental disorders of the pancreas.

## Human pancreas development

Human pancreas specification is evident at ~29 days post conception (dpc), with detection of the transcription factor pancreas and duodenal homeobox 1 (PDX1) in the dorsal foregut endoderm, which is preceded by forkhead box protein A2 (FOXA2) expression at 27 dpc [1]. In the mouse, PDX1 is detected slightly earlier, at embryonic day (E)8.5, which corresponds to ~25–27 dpc in humans [2]. By 30–33 dpc, both dorsal and ventral pancreatic buds are apparent, marked by the transcription factors PDX1, SRY-box transcription factor 9 (SOX9), GATA binding protein 4 (GATA4) and NK6 homeobox 1 (NKX6–1) [1]. At this point the buds contain multiple microlumens, which eventually form the luminal network of the branched pancreas. Both buds undergo a period of growth and branching, before fusing at ~37–40 dpc [1]. During this time there is an expansion of multipotent progenitors, marked by the presence of SOX9, GATA4 and NKX6–1. In contrast with the mouse, there is no expression of the transcription factor NKX2–2 in the early human pancreatic buds [1, 2]. With this exception, the early stages of human pancreas development follow a similar course to those in the mouse. In mice, the very first hormone-secreting cells appear at ~E9, shortly after pancreas specification (‘primary transition’). These glucagon-expressing cells are transient and do not appear to contribute to the established endocrine cell population [2]. In contrast, there is no evidence of biphasic endocrine differentiation during human development. The first insulin-positive cells appear at 8 weeks post conception (wpc) [1]. This may reflect subtle anatomical differences between species; a lack of proximity of the paired dorsal aortae to the early pancreatic endoderm might prevent the pro-endocrine signals from the aortae that are observed in mouse [2]. The hallmark of endocrine differentiation in both mice and humans is the transient expression of the transcription factor neurogenin 3 (NEUROG3). In the mouse, NEUROG3 is first expressed at E9, heralding the primary transition. Its expression coincides with more prominent endocrine differentiation in the mouse at ~E13 (‘secondary transition’) [2]. In contrast, NEUROG3 is first apparent in the human pancreas at 8 wpc, expression peaks between 10 and 14 wpc and then declines from ~18 wpc [1, 3]. It is not clear precisely when NEUROG3 is turned off in humans, but it is not detected by 35 wpc [3]. By transplanting human fetal pancreas into the mouse, NEUROG3 was detected in the graft until at least 19 weeks after transplantation [4]; both these findings suggest the period of endocrine differentiation is much longer in humans than in the mouse [5]. The downstream mechanisms by which NEUROG3 coordinates endocrine differentiation have been elucidated in the mouse [2, 6] and are overtly similar to those in humans. There are differences, such as the temporal expression of the transcription factors MafA and MafB; while MafB expression diminishes from beta cells postnatally in the mouse, its expression begins in human fetal pancreas and is sustained in adult beta cells [7, 8]. MafA expression in murine beta cells peaks soon after birth, whereas its expression continues to increase in juvenile and adult human beta cells [9, 10]. Thus, adult human beta cells express both MafA and MafB, while adult mouse beta cells only express MafA. For a comprehensive review on human pancreas development that also includes comparisons with mouse pancreas development, we refer the reader to [11].

## Transcription factors and mouse pancreas development: some outstanding questions

The expression of several pancreas transcription factors has been experimentally modified by genetic perturbations in transgenic mice. The knowledge acquired from such manipulations justifies the considerable effort needed to generate these mice [2]. *Pdx1*, for example, has been extensively studied and its deletion in the mouse leads to pancreas agenesis [12, 13]. This phenotype was first described almost 30 years ago but the underlying mechanisms by which loss of *Pdx1* has these drastic consequences remain obscure. We know that the early events that induce pancreatic budding occur in these mice, and tissue recombination experiments suggest a cell-autonomous defect in which pancreatic progenitor cells have lost competence to respond to morphogenic signals from the pancreatic mesenchyme [14]. However, we lack direct data demonstrating either decreased or absent proliferation or increased death of pancreatic progenitor cells in these mice. *Neurog3* is another well-studied transcription factor, in part due to the dramatic phenotype of *Neurog3*-deficient mice, comprising a total lack of pancreatic endocrine cells, neonatal diabetes and early postnatal death [5]. Of particular interest is the contrast between the robust phenotype in *Neurog3* null mice and the heterogenous presentation in people with *NEUROG3* loss-of-function mutations, as described below. Finally, mice lacking *Rfx6*, encoding regulatory factor X6, a target of NEUROG3, fail to generate any of the pancreatic endocrine cells except pancreatic-polypeptide-producing cells and present with small bowel obstruction, pancreas hypoplasia, albeit inconstant, and early postnatal death [15]. Of interest in the case of *RFX6* mutations in humans are the phenotypic differences compared with *NEUROG3* mutations and the persistent role of RFX6 in adult beta cells.

## Models to study the role of transcription factors in human pancreas development

Human model systems are preferable to animal models when studying human physiology and pathology to avoid the possibility of interspecies differences. However, studying the role of transcription factors in human pancreas development is more challenging because of a lack of appropriate models. In recent years, different in vitro models have been developed and used in studies aimed to increase our understanding of the impact of transcription factor perturbations on human pancreas development. First, the directed differentiation of human pluripotent stem cells (hPSCs) into pancreatic endocrine and exocrine cells has become a reliable model for normal cellular development and a valuable tool for studying pancreatic diseases (recently reviewed in [16]). For example, hPSCs have been used to study the function of specific transcription factors linked to neonatal diabetes. Either mutant cells from patients suffering from neonatal diabetes have been reprogrammed [17] or disease-specific mutations have been introduced into existing hPSC cell lines through genome editing techniques [18, 19]. In addition, the human EndoC-βH beta cell lines have been used to study the impact on beta cell function of mutations in transcription factors found in patients with neonatal diabetes. Both gain-of-function studies using gene overexpression [20] and loss-of-function studies using siRNA-mediated knockdown [21] have been reported. Other envisioned strategies using this cell line include CRISPR/Cas9 genome editing to mimic disease-specific mutations. While such 2D culture systems model cellular differentiation and are able to pinpoint developmental defects, they are, however, intrinsically unable to closely recapitulate organ growth, morphogenesis and the intercellular interactions between the endocrine and exocrine compartments. The development of human pancreatic organoids (hPOs) pushes cell culture methods into 3D and aims to more closely recapitulate in vivo differentiation. Organoids are defined as 3D structures grown from either hPSCs or tissue-resident stem/progenitor cells in which organ-specific cells spontaneously self-organise into differentiated functional cell types and at least some functions of the organ are recapitulated [22, 23]. A major advantage of hPOs is that they could be made from cells with disease-specific mutations. The closer mimicking of cellular interactions in a 3D culture of mouse pancreas progenitors has already been shown to more faithfully model exocrine and endocrine differentiation [24]. Methods for long-term culture of adult mouse pancreas organoids composed of PDX1^+^ ductal cells have also been established; however, endocrine differentiation was extremely limited in these 3D models [25]. For human cells, protocols for efficient expansion of fetal and adult duct-like tissue have been developed, however, again, subsequent differentiation into endocrine cells remains suboptimal [26, 27]. While the above-described models will surely increase knowledge on human pancreas development, caution remains warranted when interpreting these results as none of the models perfectly replicates in vivo pancreas development.

## Neonatal diabetes: a natural model to study the role of transcription factors in human pancreas development

**Fig. 1:**
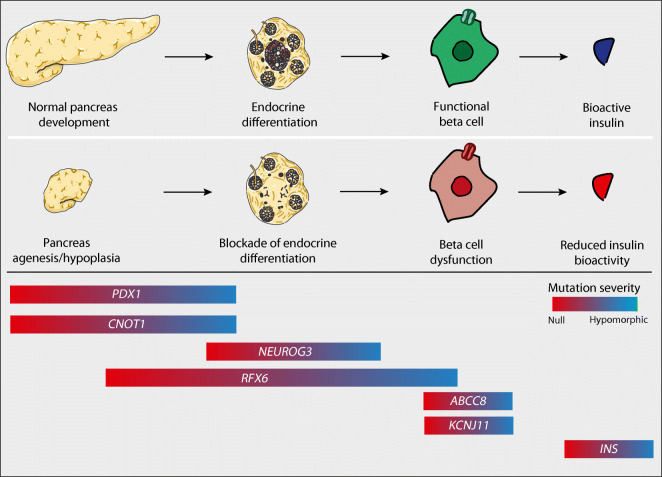
Developmental classification of permanent neonatal diabetes. Transcription factors control normal human pancreas development, from its morphogenesis, through endocrine differentiation, to beta cell development and function. Mutations in transcription factor genes such as *PDX1*, *NEUROG3* and *RFX6* are responsible for different forms of permanent neonatal diabetes, thus, neonatal diabetes could be classified according to the developmental impact of the transcription factor mutations on pancreas, islet or beta cell formation. Mutations in genes that encode proteins involved in the machinery of insulin secretion (*ABCC8*, *KCNJ11*) or insulin (*INS*) itself can also be included in such a developmental classification. Colour scale indicates mutation severity from null (red) to hypomorphic (blue). This figure was created using Servier Medical Art (https://smart.servier.com/). Servier Medical Art by Servier is licensed under a Creative Commons Attribution 3.0 Unported License. This figure is available as a downloadable slide

In the following paragraphs, we want to focus on how case reports or series of neonatal diabetes can be used to dissect the function of transcription factors during human pancreas development. Neonatal diabetes is a rare form of diabetes defined as diabetes diagnosed before 6 months of age (approximate incidence of 1:100,000 live births). Most babies with neonatal diabetes present with decreased birthweight (intra-uterine growth retardation due to fetal insulin deficiency), decreased fat reserves and low or undetectable C-peptide levels [28]. Neonatal diabetes could be classified according to the impact of its underlying mutation on aspects of pancreas development, from mutations causing complete pancreas agenesis, mutations that prevent islet formation, mutations that prevent beta cell development or cause early beta cell death, mutations that result in beta cells with defective insulin production or secretion, to mutations in the secreted insulin (Fig. 1). However, access to the pancreatic tissue of individuals with neonatal diabetes for post-mortem examination is very limited and in-depth studies of such pancreases are scarce. Somewhat over half (~57%) of affected babies have a transient, relapsing form of neonatal diabetes (TNDM) but in some of these cases diabetes may recur after a prolonged period [28, 29]. Currently, there is a list of 34 genes linked to permanent neonatal diabetes mellitus without autoimmune disease, which includes mutations in genes for 14 transcription factors, insulin itself, genes encoding proteins needed for insulin secretion (*ABCC8* and *KCNJ11*) and genes encoding proteins implicated in beta cell survival [30]. State-of-the-art genetic testing can find a genetic diagnosis for 82% of babies with neonatal diabetes [30]. In the last part of this review, we will specifically focus on permanent neonatal diabetes mellitus associated with mutations in three pancreatic transcription factors. These can be considered as human gene allelic loss models for studying the role of transcription factors in human pancreas development.

In 1997, Stoffers and colleagues first linked human pancreas agenesis to *PDX1* mutations [31] and, later, more studies confirmed this association [32, 33]. Pancreatic agenesis results in a clinical phenotype of both endocrine and exocrine deficiency. The clinical presentation of mutations in pancreatic transcription factors can vary according to the severity of the mutations and, for *PDX1*, cases presenting as isolated permanent neonatal diabetes mellitus without [34] or with subclinical exocrine insufficiency [35] have been reported. Interestingly, monoallelic loss of *PDX1* has a strong, negative impact on the generation of insulin-positive beta-like cells from hPSCs during in vitro differentiation [18, 36]. This finding is intriguing as mice that are haploinsufficient for *Pdx1* are reported to develop normally [12, 13], but become diabetic with age due to decreased beta cell function [37, 38] and increased beta cell apoptosis [39, 40]. Future studies will determine whether these results are valid in vivo or merely reflect an artefact of the in vitro system.

A block in islet and enteroendocrine cell differentiation has been linked to *NEUROG3* mutations, but the human loss-of-function phenotype is variable, especially in its pancreatic endocrine aspects. In 2006, three individuals were described with *NEUROG3* mutations giving rise to congenital malabsorptive diarrhoea with intestinal enteroendocrine cells. Unexpectedly, these individuals did not have permanent neonatal diabetes mellitus, despite their severe *NEUROG3* mutations, suggesting that an unidentified factor compensated for the lack of functional NEUROG3 in the human pancreas [41]. This hypothesis was challenged by a study reporting that the mutations in these individuals were not null but hypomorphic [42] and, more recently, biallelic null mutations in *NEUROG3* were discovered in an individual with permanent neonatal diabetes mellitus and intestinal anendocrinosis [43]. Subsequently*,* using a knockdown approach in hPSC-directed differentiation, as little as 10% *NEUROG3* was demonstrated to be sufficient for pancreatic endocrine cell differentiation [44]. Reconciliation in the field, however, was short lived as, more recently, two individuals with biallelic functionally null variants of the *NEUROG3* gene without permanent neonatal diabetes mellitus were reported [45]. The reason for the difference in pancreatic function between the *NEUROG3* null phenotype in human and mice remains poorly understood but may lie in subtle differences in the way NEUROG3 induces cell cycle arrest in pancreatic progenitors prior to endocrine differentiation [46].

Similarly, mutations in *RFX6* also block endocrine differentiation and are associated with permanent neonatal diabetes mellitus, pancreatic hypoplasia, intestinal atresia and gallbladder aplasia or hypoplasia [15, 47]. These phenotypic differences compared with *NEUROG3* deficiency are attributed to earlier expression of *RFX6* in the early gut endoderm [15]. Enteric anendocrinosis, as documented in the case of *NEUROG3* loss, has not been studied in detail for *RFX6* loss in humans [48]. When *RFX6* was deleted in hPSCs it decreased the efficiency with which pancreatic progenitors were generated from hPSCs [18], which is in line with the reduced size of the pancreas in some of the *Rfx6*-deficient mice and in people with biallelic *RFX6* mutations [15, 49]. Apart from its role in pancreas development, RFX6 is important for the functioning of adult beta cells. In the human beta cell line EndoC-βH2, RFX6 regulates insulin gene (*INS*) transcription, insulin content and secretion and its knockdown decreased the activity of Ca^2+^ channels important for insulin exocytosis [21]. Mouse studies that established a regulatory role for RFX6 in islet development [50] have also shown that RFX6 is essential for maintaining the functional identity of adult beta cells, as it suppresses disallowed genes such as *Ldha*, *Slc16a1*, *Pdgfra* and *Igfbp4* [48]. This effect on beta cell function has also been found in humans, as heterozygous truncating variants of RFX6 were found to cause a form of maturity-onset diabetes of the young (MODY) with reduced penetrance [51].

## Conclusions

Pancreas development is regulated by hierarchical gene regulatory networks in which some transcription factors implicated in the pathology of permanent neonatal diabetes mellitus are key players. While many developmental steps are similar in mice and humans, there are intriguing differences, some of which we have highlighted. It is important to note that most of what we know about mammalian pancreas development stems from mouse genetics. The study of human pancreas development has benefited from mouse data, but also from model systems such as directed differentiation of hPSCs and human beta cell lines. Modelling human disease or development, however, is still modelling; while clinical cases of neonatal diabetes provide us with direct evidence of the impact of mutations in transcription factors, albeit often only evidence of the outcome and not of the process we are trying to understand. Finding a genetic cause for most forms of neonatal diabetes is currently feasible, but the genetics of some syndromic forms remain unclear and are thus a challenge for the future. The recent report of a mutation in the transcription regulator *CNOT1* as a cause of pancreas and gallbladder agenesis [52, 53], a regulator that had not been previously identified as important for mouse pancreas development, shows that the natural model of human disease still has interesting insights to offer [54, 55]. The scientific progress in our understanding of neonatal diabetes has increased our understanding of more common forms of diabetes, but hopefully they too will benefit from therapeutic advances. In the past, transcription factors were not considered targets for drug development, but the increased understanding of diseases and transcriptional regulation is enabling the development of such therapeutic strategies in other diseases [56]. We cannot expect such strategies to be useful for people with early and severe developmental defects, such as complete pancreas agenesis, but they may be able to target less severe forms of neonatal diabetes with mainly terminal differentiation defects. Neonatal diabetes already has success stories, but we are keen to add new ones to the good track record.

## Electronic supplementary material

Figure slide (PPTX 192 kb)

## Abbreviations

ABCC8: ATP-binding cassette transporter subfamily C member 8
dpc: Days post conception
E: Embryonic day
GATA4: GATA binding protein 4
hPO: Human pancreatic organoid
hPSC: Human pluripotent stem cell
NEUROG3: Neurogenin 3
NKX6–1: NK6 homeobox 1
PDX1: Pancreatic and duodenal homeobox 1
RFX6: Regulatory factor X6
SOX9: SRY-box transcription factor 9
wpc: Weeks post conception
