# Supplementary material for: Transcription factors that shape the mammalian pancreas
Source: Diabetologia. 2020 Sep 7;63(10):1974–80. doi: 10.1007/s00125-020-05161-0 (PMC7476910; doi:10.1007/s00125-020-05161-0)
Supplement: Supplementary file 1 — (PPTX 192 kb) [file 125_2020_5161_MOESM1_ESM.pptx]

## Slide 1
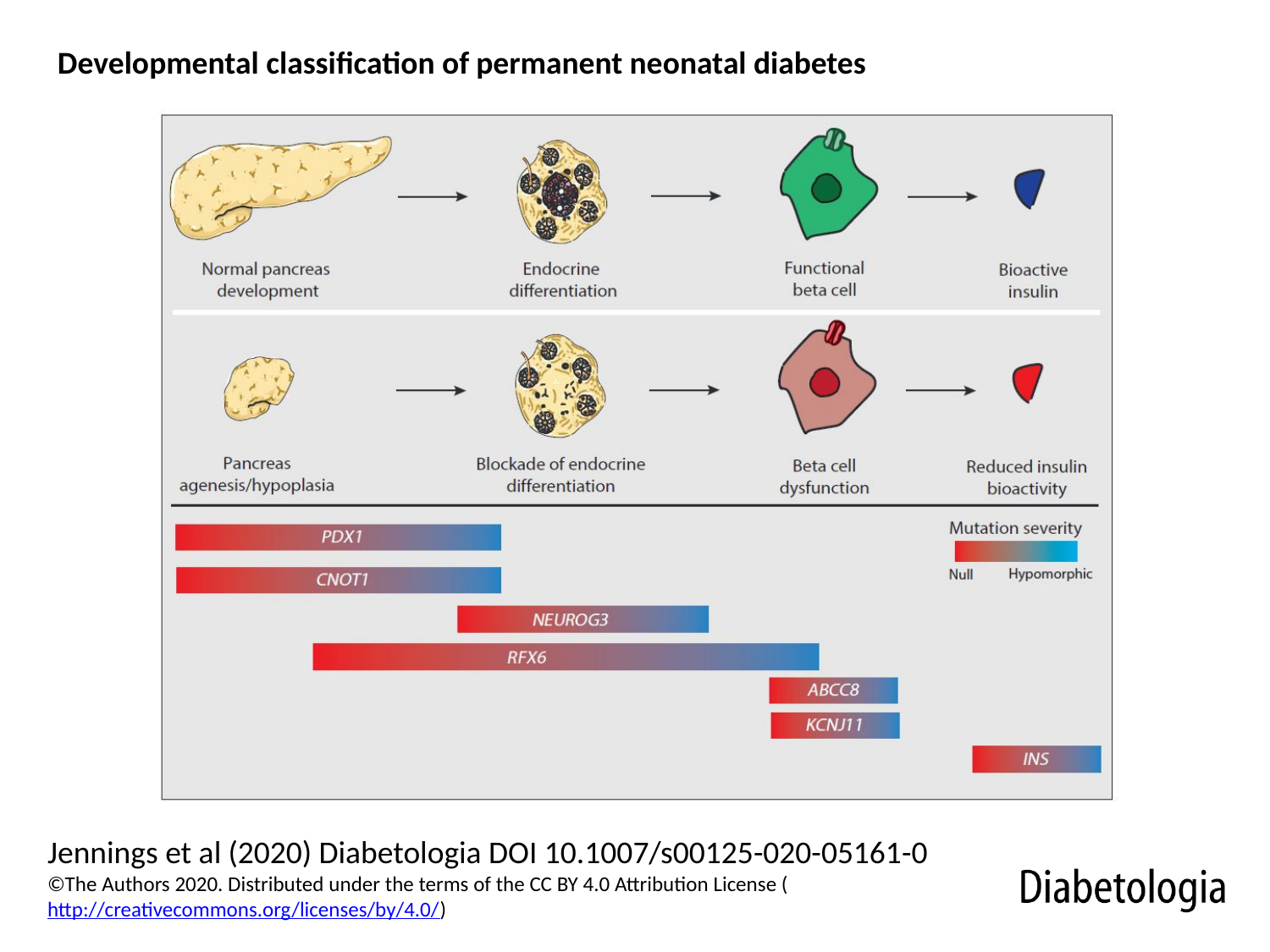

Developmental classification of permanent neonatal diabetes
Jennings et al (2020) Diabetologia DOI 10.1007/s00125-020-05161-0
©The Authors 2020. Distributed under the terms of the CC BY 4.0 Attribution License (http://creativecommons.org/licenses/by/4.0/)
